# The Fetal Type of Posterior Cerebral Artery

**DOI:** 10.3390/medicina59020231

**Published:** 2023-01-26

**Authors:** Ana-Maria Davidoiu, Dragoş Ionuţ Mincă, Mugurel Constantin Rusu, Sorin Hostiuc, Corneliu Toader

**Affiliations:** 1 Faculty of Medicine, “Victor Babeş” University of Medicine and Pharmacy, RO-300041 Timișoara, Romania; 2 Division of Anatomy, Department 1, Faculty of Stomatology, “Carol Davila” University of Medicine and Pharmacy, RO-020021 Bucharest, Romania; 3 Division of Legal Medicine and Bioethics, Faculty of Stomatology, “Carol Davila” University of Medicine and Pharmacy, RO-020021 Bucharest, Romania; 4 Division of Neurosurgery, Department 6–Clinical Neurosciences, Faculty of Medicine, “Carol Davila” University of Medicine and Pharmacy, RO-020021 Bucharest, Romania; 5 Clinic of Neurosurgery, “Dr. Bagdasar-Arseni” Emergency Clinical Hospital, RO-041915 Bucharest, Romania

**Keywords:** internal carotid artery, cerebral artery, vertebral artery, basilar artery, posterior communicating artery

## Abstract

*Background and Objectives*: Anatomical variations of the arterial circle of Willis (cW) are common. A posterior cerebral artery (PCA) fed mostly or exclusively from the internal carotid artery is a fetal PCA (FPCA), partial (p-FPCA), or full/complete (f-FPCA), respectively. Because FPCA occurs in different anatomical configurations of the cW sides, we aimed to document in detail these morphological possibilities of FPCA within the cW. *Materials and Methods*: FPCAs were documented on a retrospective set of 139 computed tomography angiograms. *Results*: FPCAs were found in thirteen cases, nine males and four females. In 7/13 cases there were two modified sides of the cW. In 5/13 cases there were three altered sides of the cW. Another case with FPCA showed four altered sides of the cW. In 10/13 cases, FPCA was unilateral and in the other three cases it was bilateral. Compared to the overall group, unilateral p-FPCAs were found in 1.43%, while unilateral f-FPCAs were found in 5.75%. A bilateral p-FPCA-f-FPCA combination was found in 0.71% and a bilateral f-FPCA-f-FPCA combination occurred in 1.43%. An anatomically isolated ICA was found in just one case with bilateral f-FPCA (0.71%). In 7/13 FPCA cases there were arterial variants exclusively in the posterior cW. In the other 6/13 FPCA cases, there were variants in both anterior and posterior circulation. There were no statistically significant associations of FPCA with sex or age. The higher prevalence of right-sided FPCA was not statistically significant. *Conclusions*: Anatomical assessments of cW should be performed on a case-by-case basis, as they may correspond to different cW morphologies.

## 1. Introduction

The circle, or polygon, of Willis (cW) is one of the most famous eponymous structures in human anatomy [1]. Anatomic variations in the cerebral arterial circle of Willis are the rule, not the exception [2]. Studies report a classical cW to be present in anywhere from 4.8% to 85.4% of the population, as was recently reviewed [3]. Seemingly, the distributions of the variations of cW do not differ in different populations [4]. Variants of the cW may impede correct identification of ischemic lesion patterns and stroke etiology [5].

**Figure 1:**
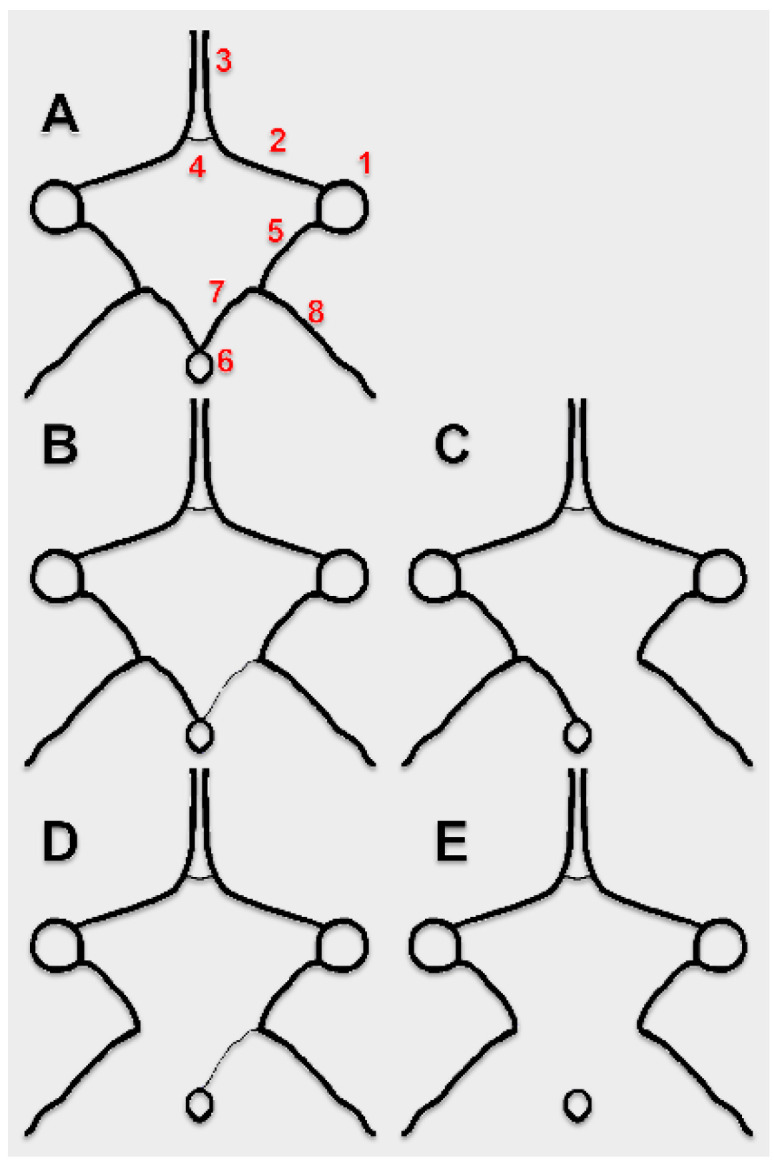
Superior views of the circle of Willis. (**A**). Complete circle of Willis. (**B**). Partial fetal posterior cerebral artery (p-FPCA), with hypoplastic P1 segment of the PCA. (**C**). Full/complete fetal posterior cerebral artery (f-FPCA), with aplastic P1 segment of the PCA. (**D**). Bilateral fetal posterior cerebral artery, p-FPCA+f-FPCA combination; (**E**). Bilateral fetal posterior cerebral artery, f-FPCA+f-FPCA combination. Other possible variations of the sides of the circle of Willis are not considered here. 1. ICA; 2. A1 segment of the ACA; 3. A2 segment of the ACA; 4. AComA; 5. PComA; 6. BA; 7. P1 segment of the PCA; 8. P2 segment of the PCA.

The “textbook” type of cW is the polygon with nine sides, a nonagon: the anterior communicating artery (AComA) uniting the anterior cerebral arteries (ACAs), the A1 (precommunicating) segments of the ACAs, the internal carotid arteries (ICAs) they emerge from, the posterior communicating arteries (PComAs), and the P1 (precommunicating) segments of the posterior cerebral arteries (PCAa) which leave the basilar artery (BA) [6,7] (Figure 1). In the adult type, the P2 (postcommunicating) segment of the PCA continues the P1 segment. 

The arteries of interest to the neuro-ophthalmologists are the ophthalmic and central retinal arteries anteriorly and the PCA posteriorly [8]. Three types of configuration of the cW were discussed, as related to the P1 segment of the PCA: (a) the adult configuration in which the P1 is larger than the PComA, which is not hypoplastic; (b) the transitional configuration in which the PCA and P1 have the same diameter, and (c) the fetal or embryonic configuration in which the diameter of the P1 is smaller than the diameter of the PComA [9]. 

In fetal types of PCA (FPCA) the P2 segment derives from the ICA. The P1 segment could be either hypoplastic (partial FPCA, p-FPCA) or absent (full FPCA, f-FPCA) (Figure 1), according to the definition of van Raamt et al. (2006) [10]. Patients with a partial FPCA could be more prone to develop ischemic strokes [11].

Few studies are solely focused on the anatomy of PCA [12]. In a recent study, the fetal type of PCA (FPCA) was found only unilaterally in 5.6% of 231 cases [13]. van Raamt reviewed different studies and found that bilateral FPCAs occur in up to 9% of cases [10].

As the FPCA occurs in different anatomical combinations with variant sides of the cW we aimed to anatomically document such variants on a retrospective lot of computed tomography angiograms (CTAs), and to test whether the FPCA variant is related to either sex or side. 

## 2. Materials and Methods

We conducted a retrospective study on 147 randomly selected computed tomography angiograms (CTAs) to evaluate the arteries of the cW. Inclusion criteria were age of subjects (>18 years), adequate quality of angiograms, no pathologic processes distorting the cW, and no previous history of surgery on the cW. Exclusion criteria were pathological processes distorting the arterial anatomy of the cW, and degraded or incomplete computed tomography scans. After applying these criteria, we retained 139 angiograms from 83 male and 56 female Caucasian subjects aged between 58 and 74 years. All subjects gave their informed consent for inclusion before they participated in the study. The research was conducted following principles from The Code of Ethics of the World Medical Association (Declaration of Helsinki). The Ethics Committee of the “Dr. Bagdasar-Arseni” Emergency Clinical Hospital approved the study (approval no. 2093/01.03.2022). 

The CTAs were performed with a 32-slice scanner (Siemens Multislice Perspective Scanner), with a 0.6 mm collimation and a reconstruction of 0.75 mm thickness with 50% overlap for a multiplanar maximum intensity projection (MIP) and three-dimensional volume rendering (3D-VR) technique, as described previously [14]. The cases were documented using Horos for iOS (Horos Project). Evaluations of the presence and subtypes of FPCAs were independently performed by an experienced anatomist (author #3) and a neurosurgeon (author #5). The positive results were identical and were validated by each author. 

In cases with FPCA, the following variables were documented: (1) the number of variant sides (hypoplastic/aplastic) of the cW, as referred to the normal pattern; (2) the individual morphology of the cW; (3) the uni- or bilateral presence of an FPCA; (4) the subvariant of the FPCA, either p-FPCA, or f-FPCA (Figure 1). As the anterior choroidal artery does not participate in the cW, it was not evaluated on CTAs. 

Statistical analysis was performed using SPSS v.29 for MacOS. We used descriptive statistics to test associations between qualitative variables, we used the Pearson Chi^2^ test and we used ANOVA to compare means between subgroups. A “*p*” value below 0.05 was considered statistically significant. For comparison of FPCA lateralization (right side vs. left side) in cases with unilateral FPCA, we performed a binomial test.

## 3. Results

From the general lot of 139 cases, 13 cases with FPCA (9.35%) were documented. Of these cases, nine were male (69.23%) and four were female cases (30.77%) (Table 1). 

In 10/13 cases (76.92%) the FPCA was unilateral, in 7/10 cases on the right side, and in 3/10 cases on the left side. In three other male cases (23.07%) we found bilateral FPCAs (Table 1).

**Figure 2:**
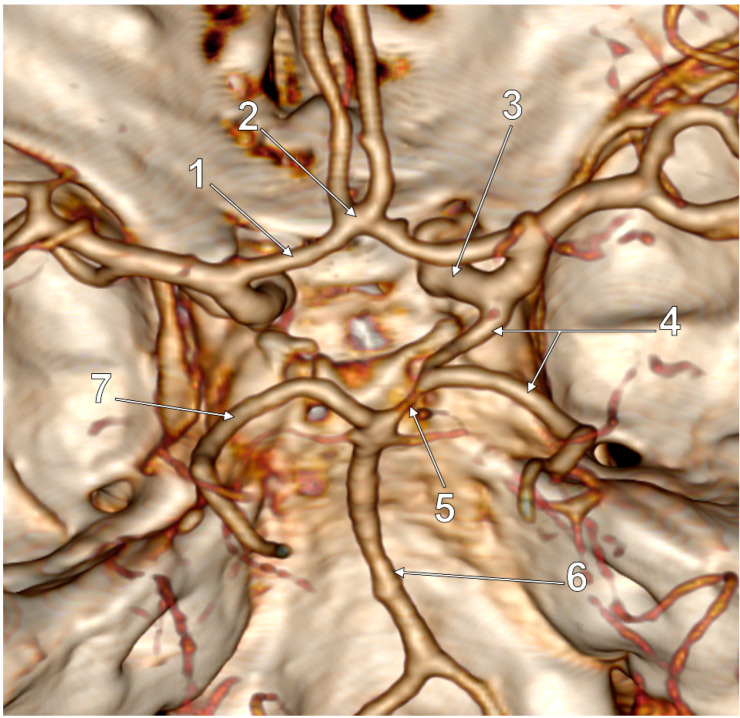
Postero-superior view of the cW. Unilateral partial FPCA (p-FPCA). Two variant sides of the cW: hypoplastic right P1 and aplastic left PComA. 1. A1 segment of the left ACA; 2. AComA; 3. Right ICA; 4. Right p-FPCA; 5. Right P1 hypoplasia; 6. BA; 7. Left PCA.

**Figure 3:**
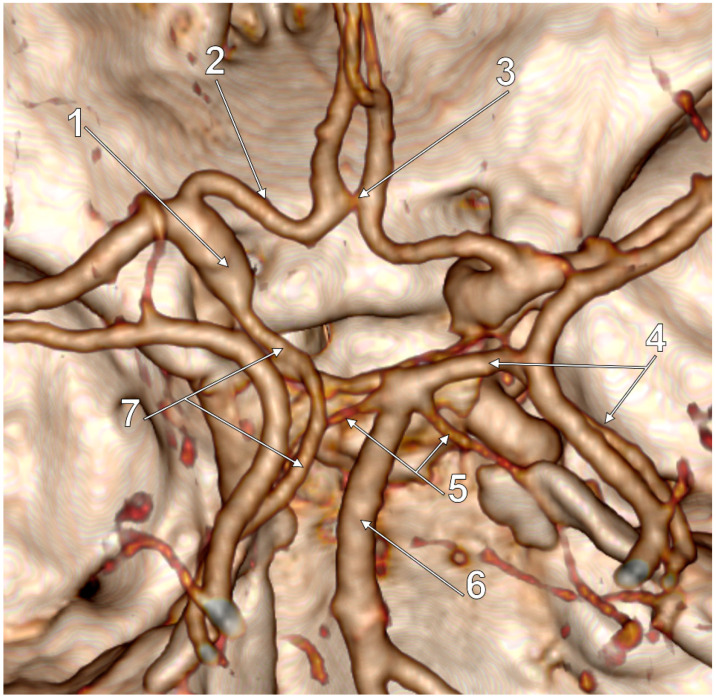
Postero-superior view of the cW. Unilateral full/complete FPCA (f-FPCA). Two variant sides of the cW: aplastic left P1 and aplastic right PComA. 1. Left ICA; 2. A1 segment of the left ACA; 3. AComA; 4. Right PCA (P1+P2); 5. Superior cerebellar arteries; 6. BA; 7. Left f-FPCA.

**Figure 4:**
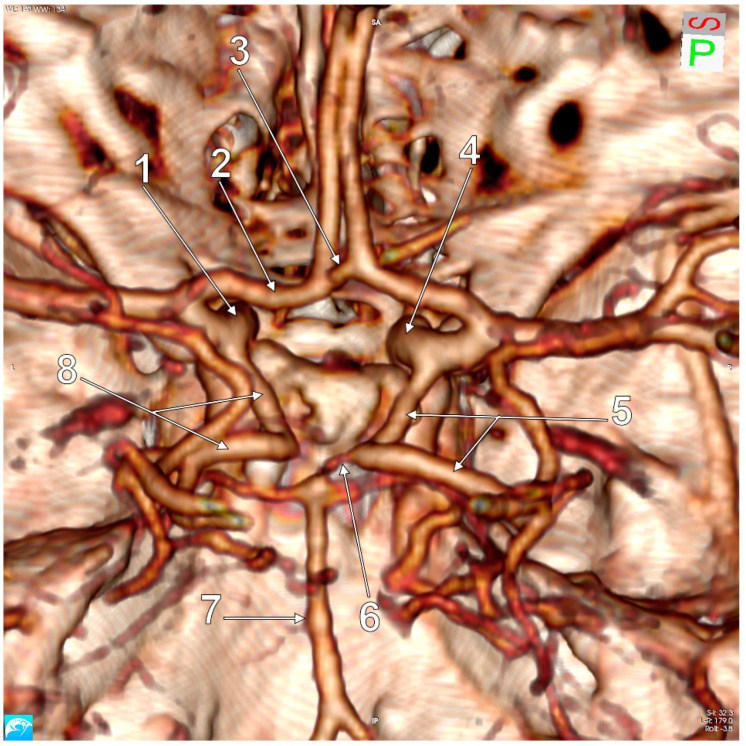
Postero-superior view of the cW. Bilateral FPCAs, partial and full/complete (p-FPCA+f-FPCA). Two variant sides of the cW: hypoplastic right P1 and aplastic left P1. 1. Left ICA; 2. A1 segment of the left ACA; 3. AComA; 4. Right ICA; 5. Right p-FPCA; 6. Right P1 hypoplasia; 7. BA; 8. Left f-FPCA.

**Figure 5:**
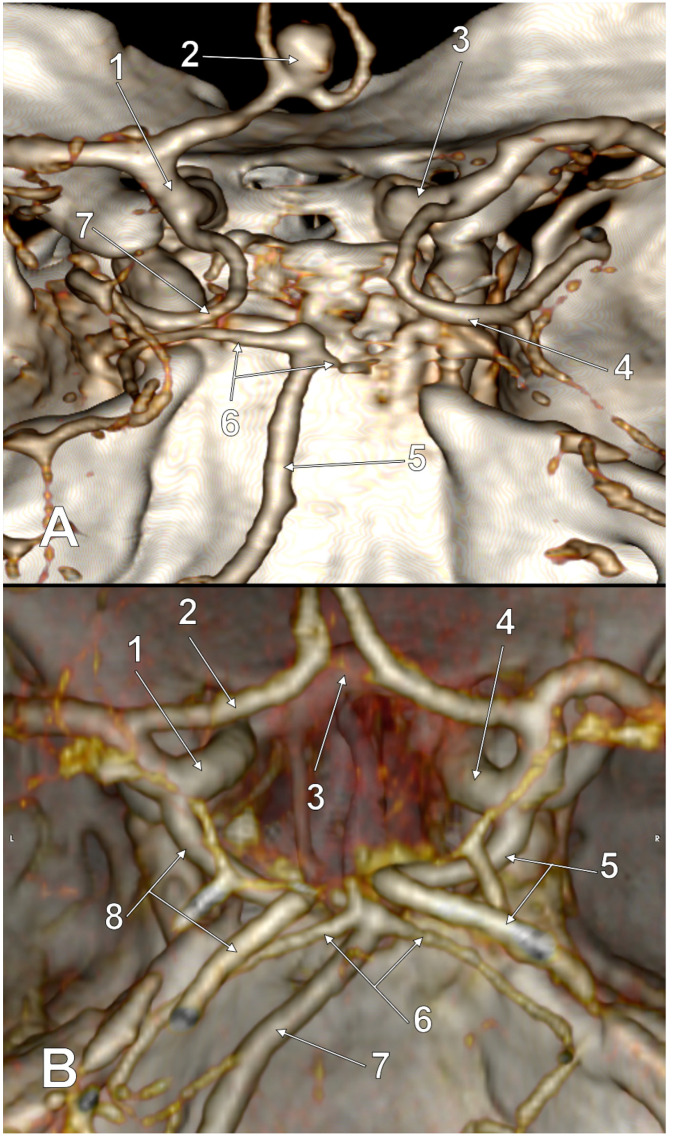
Postero-superior views of the cW. Bilateral full/complete FPCAs (f-FPCA+f-FPCA). Three variant sides of the cW. (**A**) Aplastic right A1 and bilateral aplasia of P1. 1. Left ICA; 2. AcomA aneurysm; 3. Right ICA (isolated ICA); 4. Right f-FPCA; 5. BA; 6. Superior cerebellar arteries; 7. Left f-FPCA. (**B**) Hypoplastic AComA and bilateral aplasia of P1. 1. Left ICA; 2. A1 segment of the left ACA; 3. Hypoplastic AComA; 4. Right ICA; 5. Right f-FPCA; 6. Superior cerebellar arteries; 7. BA; 8. Left f-FPCA.

In the general lot, unilateral p-FPCAs were found in two male cases (2/139, 1.43%) (Figure 2). Unilateral f-FPCAs were found in four male and four female cases (8/139, 5.75%) (Figure 3). One male case had the p-FPCA+f-FPCA bilateral combination (1/139, 0.71%) (Figure 4). The f-FPCA+f-FPCA bilateral combination was found in two male cases (2/139, 1.43%) (Figure 5), thus the bilateral ICA supply of the PCA territory (Table 1).

Of the 13 cases with FPCA, four males and three females had two sides of the cW altered from normal morphology (53.84%). In five other cases, four males and one female, the cW had three sides altered from normal morphology (28.46%). Only one male case had four sides of the cW altered (7.69%) (Table 2).

**Figure 6:**
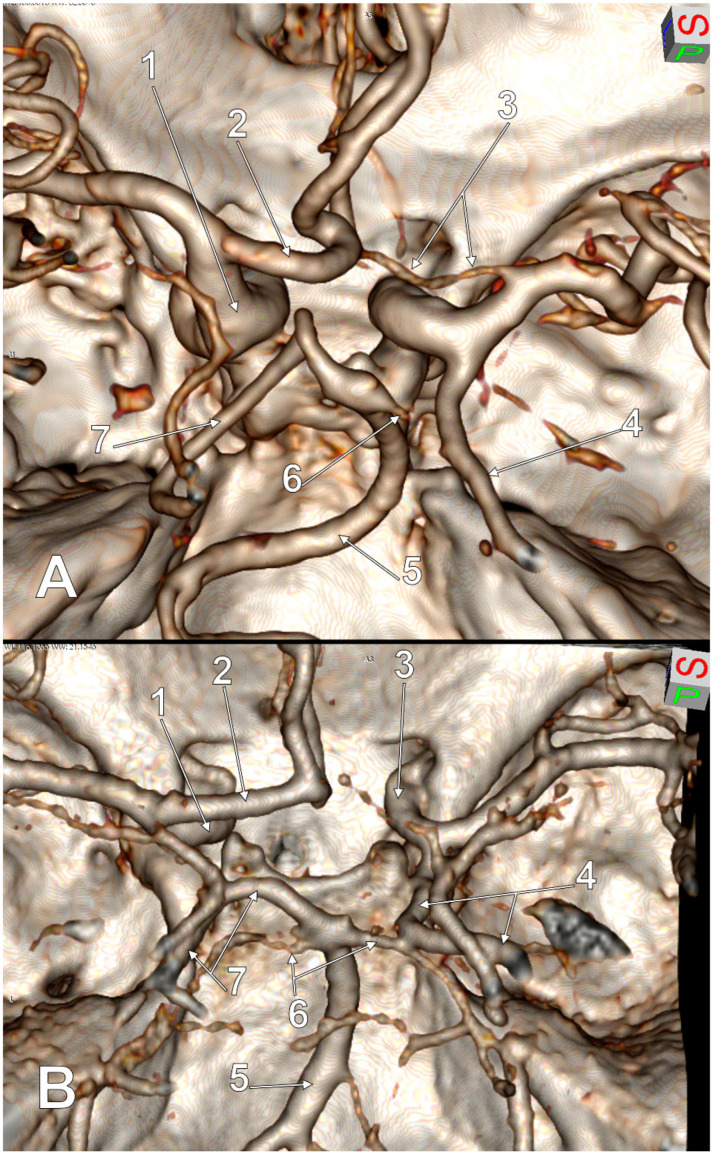
Postero-superior views of the cW. Unilateral full/complete FPCAs (f-FPCA). Three variant sides of the cW. (**A**). Hypoplastic right A1, ipsilateral aplasia of P1, contralateral aplasia of PComA. 1. Left ICA; 2. A1 segment of the left ACA; 3. Hypoplastic A1 segment of the right ACA; 4. Right f-FPCA; 5. BA; 6. Right superior cerebellar artery; 7. Left PCA. (**B**). Right aplasia of A1 and P1, left aplasia of PComA. 1. Left ICA; 2. A1 segment of the left ACA; 3. Right ICA; 4. Right f-FPCA; 5. BA; 6. Superior cerebellar arteries; 7. Left PCA.

**Figure 7:**
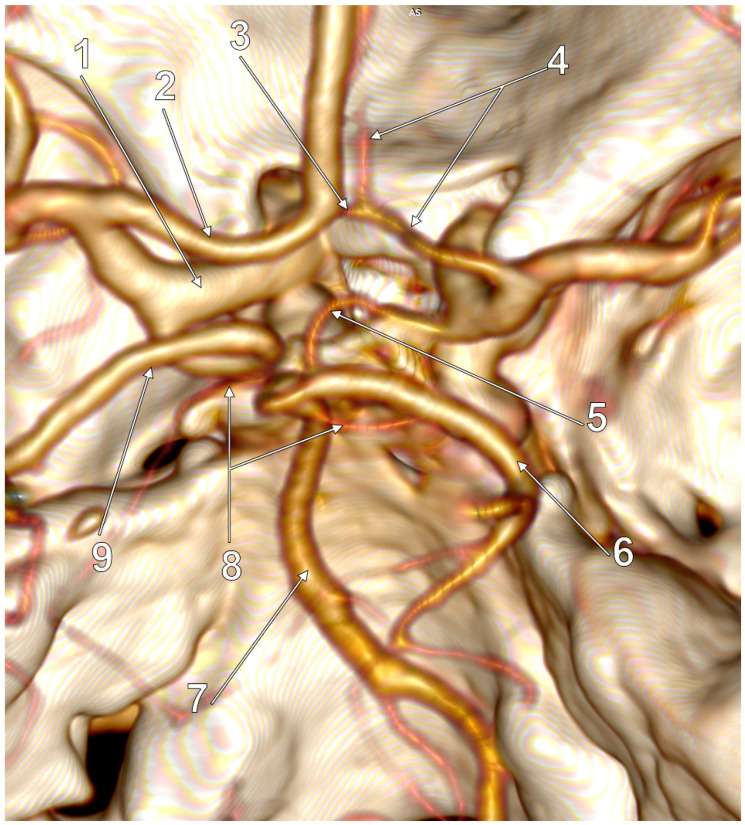
Posterior view of the cW. Full/complete left FPCAs (f-FPCA). Four variant sides of the cW: hypoplastic right ACA, hypoplasia of AComA, hypoplastic PComA and aplastic contralateral P1. 1. Left ICA; 2. A1 segment of the left ACA; 3. Hypoplasia of AComA; 4. Hypoplastic right ACA; 5. Hypoplastic right PComA; 6. Right PCA leaving the left side of the BA distal end; 7. BA; 8. Superior cerebellar arteries; 9. Coiled left f-FPCA.

The different subvariants of FPCA were found in different anatomical variants of the cW regarding the number of modified sides and the general morphology of the cW (Table 2, Figure 2, Figure 3, Figure 4, Figure 5, Figure 6 and Figure 7). Seven of the 13 cases with FPCA had only posterior sides of the cW modified (Figure 2, Figure 3 and Figure 4) and 6/13 FPCA cases had variants in both anterior and posterior parts of the cW.

**Figure 8:**
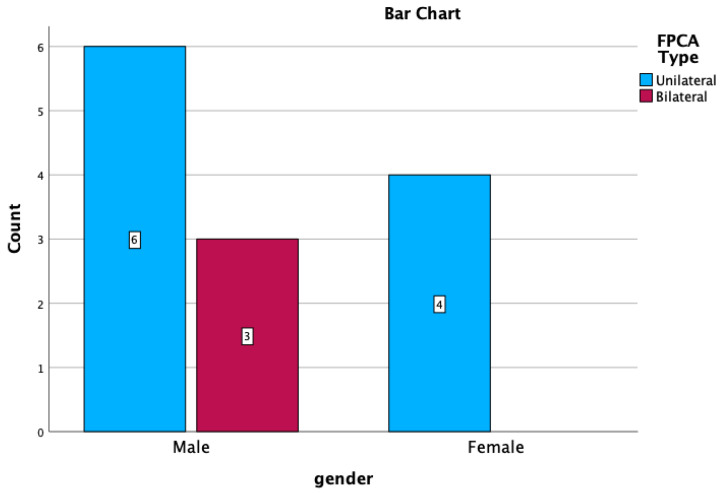
Distribution of uni/bilateral FPCA depending on the sex.

There are no statistically significant differences regarding the distribution of uni-/bilateral variants depending on sex (Pearson Chi^2^ = 1.051, *p* = 0.205, see Figure 8), 

The mean age for the unilateral variant was 67.4+/−7.3 years, while for the bilateral variant it was 62.33+/−7.5 years. The differences were not statistically significant (ANOVA, F = 1.091, *p* = 0.319).

The binomial test indicated that the proportion of right side FPCA of 0.70 was higher than the expected 0.50, but the difference compared to the FPCA on the left side is not statistically significant (*p* = 0.344).

## 4. Discussion

The development of the occipital and temporal lobes, especially in their basal portions, follows that of the frontal lobe [15]. This conditions the growth of the PCAs that are the last to form [15]. Seemingly, the variations of the posterior portion of the cW do not depend on the previous condition of the BA [15]. In embryos of 28–30 days the cranial and caudal divisions of the primitive ICA are established [16]. The latter anastomose with the longitudinal neural arteries that, in turn, will further form the BA [16,17]. The primitive PComA and the stem of the PCA derive from the caudal branch of the primitive ICA [16]. The primitive PComA normally regresses in caliber as the vertebrobasilar system develops [18]. If the primitive PComA does not regress, the main supply of the PCA will be provided from the ICA via the PComA, thus by an FPCA [16]. In cases of FPCA, endovascular approaches could benefit from a carotid, and not vertebrobasilar, route. Neurosurgeons have to avoid occluding an FPCA while treating ICA-PComA aneurysms, to avoid ischemic events in the PCA territory [16]. An FPCA could provide collateral circulation to the posterior brain after an embolus had occluded the top of the BA [19]. Ischemic events, however, also depend on the collateral anastomoses of the leptomeningeal vessels, allowing, or not, a vertebrobasilar supply of the ICA territory and vice versa [10]. 

Morphologically, an FPCA is completely different from an accessory or, respectively, a replaced PCA [20]. An FPCA supplies the entire territory of a PCA, while an accessory PCA is a hyperplastic anterior choroidal artery just partly supplying the territory of the PCA [20]. A replaced PCA is an anterior choroidal artery sending off all the branches of the PCA [21].

In the present study we did not find significant statistical associations between FPCA and sex or age. In 231 cases 63 cases (27.3%) with PComA hypoplasia and 13 cases (5.6%) with FPCA were found [13]. The statistical association between the hypoplasia of PComA in relation to sex and side was found to be highly significant [13]. However, no statistical significance between the FPCA and sex was found in that study [13]. A different study found that FPCA is significantly more common in women (*p* < 0.001) [22]. A recent study demonstrated that the prevalence of FPCA was similar in ischemic stroke patients (31%) and unselected patients (32%); unilateral FPCA was significantly more frequent on the right than on the left side in both groups (15% right vs. 8% left) [23]. Although we found right-sided FPCAs in 7/10 cases with unilateral FPCA here, the difference was not found to be statistically significant, probably due to the small size of the general lot. 

Interestingly, a statistically significant association was found between the absence of FPCA and basilar tip aneurysms, justified by a reduced hemodynamic stress in cases with FPCA [24]. So, the FPCA could be regarded as a protective variant for formation of basilar tip aneurysms [24].

A different, exploratory study found p-FPCAs in 15.1 % and f-FPCAs in 9.5% of cases [25]. Of f-FPCAs, 45.1% were on the right side, 35.3% were on the left side, and 19.6% were bilateral [25]. Of p-FPCAs, 43.9% were on the right side, 23.3% were on the left side, and 32.9% were bilateral [25]. Bilateral p-FPCA/f-FPCA combinations were found in 8/536 cases [25]. Patients with f-FPCA were older (*p* = 0.025) and more commonly female (*p* = 0.013) when compared to patients with p-FPCA or no FPCA [25]. A later study on 202 patients found that the odds of having ischemic strokes in patients with f-FPCAs and p-FPCAs were 1.448 (*p* = 0.391) and, respectively, 3.027 (*p* = 0.0307) [11].

A single-side variant of the cW leading to an FPCA was not found in the present study. Such single-side variant of the cW, of aplastic P1 segment, was found by Coulier (2021) in 2.55% of cases [26]. However, Iqbal (2013) found single-side variants of the cW in 24% of cases [27]. The FPCA could be rarely an isolated variation of the cW. Therefore, other variations of the cW should be considered and documented when an FPCA is found.

Klimek-Piotrowska et al. (2016) studied 100 cW and found typical configurations in just 27% of the specimens [28]. In 73 atypical cW the authors of the study found a single side variant in 27 cases, two variant sides in 34 cases, three variant sides in 11 specimens and a cW with five variant sides [28]. They did not encounter any cW with four variant sides [28], such as the one we report on here. The authors found the unilateral P1 hypoplasia in 4% of cases, alone or in different combinations with other variants of the cW sides. We did not find explicitly in their results whether, or not, any FPCA was found. 

Jinkins (2000) listed different possibilities of variation of the cW, one of these being the FPCA, partial or full/complete, that occurs, according to that author, in 20% of cases [29]. We found it in just 9.35% of cases. van Raamt et al. (2006) documented the FPCA prevalence in different publications as varying from 3% to 36% [10]. Kovac et al. (2014) found FPCAs in 37% of cases [30]. Differences in detection method and definition of FPCA may account for the variance in reported prevalence of FPCA [31].

Among the most common variations of the cW listed by Osborn (1999) were the FPCA with hypoplasia of P1 (15–22%), and, respectively, the anatomically isolated ICA, with FPCA and absent A1 [2]. We unilaterally found such an anatomically isolated ICA in just one case with bilateral f-FPCA (0.71%). 

Coulier (2021) documented and classified the embryologic variations of the P2 postcommunicating segment of the PCA supply as follows: (a) agenesis of the PComA; (b) hypoplasia of the PComA; (c) intermediate type of FPCA, in which the caliber of P1 and PComA are rather similar; (d) p-FPCA, and (e) f-FPCA [26]. This author found unilateral p-FPCAs in 8.8% of cases, and bilateral p-FPCAs in 2.35% of cases [26]. Unilateral f-FPCAs were found in 9.4% of cases, while bilateral f-FPCAs were found in 2.55% of cases [26]. Seemingly, in that lot of study the prevalence of FPCAs was greater than in our lot. 

Among 702/1131 ischemic stroke patients only 21.8% had variants in both anterior and posterior cW and a unilateral FPCA was found in 137/1131 cases (12.11%) [32]. A different study found unilateral FPCAs in 25.6% of cases and bilateral FPCAs in just 7.7% of cases [5]. We found only 4.31% of the general lot of 139 cases with variants in both anterior and posterior parts of the cW and unilateral FPCAs were found in 7.18% of cases. 

Different authors indicate that an absent P1 segment is very uncommon [2,33,34]. Riggs and Rupp (1963) failed to find a single case of actual absence of any side of the cW in 994 cases [35]. We found absent P1 segments in 7.19% of the total cases. 

Other studies documented the anatomical variations of the cW either in its anterior part, or in the posterior one [9,33,36,37,38,39]. Lippert and Pabst (1985) classified the variations of the posterior part of the cW without distinguishing between p-FPCAs and f-FPCAs [36]. They indicated three variants of FPCA, as follows. In 10% the FPCA occurs due to an absence or hypoplasia of the P1 segment, this being the only variant side of the posterior cW. In 5% there are bilateral FPCAs. In the other 10% the unilateral FPCA is combined with a contralateral absent PComA, which makes two variant sides of the posterior cW, the P1 segment of the PCA and the contralateral PComA. Here we found unilateral FPCAs, partial and complete, in 7.18% of cases, and bilateral FPCAs in 2.14% of cases. Van Overbeeke et al. (1991) studied 100 adult cW and found a fetal configuration in 14%; the authors also investigated 53 brains of fetuses and infants and reached the conclusion that the variations of the posterior cW are the result of developmental modifications [9]. Krabbe-Hartkamp et al. (1998) studied the cW in 150 subjects using MR angiography and found unilateral FPCAs in 25% of cases and bilateral FPCAs in 7%. These authors represented schematically the subvariants of FPCAs [37], which mostly correspond to our findings. Li et al. (2011) found FPCAs in just 17 of 160 cases (10.62%), 15 being p-FPCAs and 2 f-FPCAs [38]. Kapoor et al. (2008) examined 1000 autopsied specimens, but the different types of arterial variations were mostly studied one-by-one. Multiple variations of the cW were found in 7.4% of cases [40]. Hypoplastic P1 were found in 106 brains, of which 14 were bilateral [40]. No aplastic P1 segments of the PCA were found [40]. Enyedi et al. (2021) studied the cW using MR angiography. They found unilateral and bilateral FPCAs in 20 cases from a total lot of 126 patients but details regarding whether these FPCAs were either partial or full/complete are mostly lacking [39]. Rothberg et al. (1977) reported a case with an FPCA continued directly as the only major branch of the right ICA [41]. This appears as a rare anatomic possibility. 

An incomplete posterior cW is associated with migraine [42,43]. The frequency of PCA territory infarcts is not more than 5–10% [44]. The territory of the PCA includes the paramedian midbrain, the medial and postero-lateral thalamus, the occipital lobe, and variable major areas of the parietal and posterior temporal lobes [44]. The variability of PCA infarction depends on a BA or ICA supply of it [44]. If an FPCA is present, embolization from the ICA causing occipital infarction is possible [8]. Given its small caliber, the PComA may act as a filter, preventing the passage of emboli from the ICA to the PCA [45]. Faster perfusion transit times are seen for parameters sensitive to macrovascular transit effects ipsilateral to an FPCA in proportion to the degree of arterial asymmetry [46]. Knowledge of this normal variation is critical in the interpretation of perfusion studies because anatomical asymmetry of PCA could mimic cerebrovascular pathology [46]. On the other hand, when an FPCA is present, thromboembolism in the anterior circulation may result in paradoxical PCA territory infarction, sparing, or not, the territories of the middle or the anterior cerebral arteries [31]. An FPCA should be suspected in cases of simultaneously anterior and posterior evidence of ischemic lesions in the same hemisphere [45]. In particular, patients with hemodynamically significant carotid occlusive disease and ipsilateral FPCAs may lack the capacity to recruit anastomoses from collateral circulation within the cW [45]. When other sides of the cW are absent or hypoplastic, such collateral recruitment is even more difficult.

## 5. Conclusions

Anatomical evaluations of the cW should be performed on a case-by-case basis, as the side variations of that circle in individuals are unpredictable. If an FPCA, a variant we found in just 9.3%, is encountered, this could not predict other anatomical variations of the circle of Willis.

## Figures and Tables

**Table 1:** Prevalence of different variants of FPCA (*n* = 13). M: male, F: female.

GENDER	UNILATERAL		BILATERAL	
	*R*	*L*	*p-FPCA + f-FPCA*	*f-FPCA + f-FPCA*
M	4 (30.76%)	2 (15.38%)	1 1 (7.69%)	2 (15.38%)
F	3 (23.07%)	1 (7.69%)	-	-

**Table 2:** Variant sides of the cW in cases with FPCA. M: male, F: female.

No. of Cases, Sex	Number of Variant Sides of the cW	Morphology of the cW	Subvariant of FPCA	Figure
2 M	2	- unilateral aplasia of PComA - contralateral hypoplasia of P1	p-FPCA	2
1 M, 3 F	2	- unilateral aplasia of PComA - contralateral aplasia of P1	f-FPCA	3
1 M	2	- unilateral aplasia of P1 - contralateral hypoplasia of P1	p-FPCA+f-FPCA	4
1 M, 1 F	3	- unilateral hypoplasia of A1 - ipsilateral aplasia of P1 - contralateral aplasia of PComA	f-FPCA	6A
1 M	3	- unilateral aplasia of A1 - bilateral aplasia of P1	f-FPCA+f-FPCA	5A
1 M	3	- hypoplasia of AComA - bilateral aplasia of P1	f-FPCA+f-FPCA	5B
1 M	3	- unilateral aplasia of A1 - ipsilateral aplasia P1 - contralateral aplasia of PComA	f-FPCA	6B
1 M	4	- unilateral hypoplasia of ACA (A1+A2) - hypoplasia of AComA - ipsilateral hypoplasia of PComA - contralateral aplasia of P1	f-FPCA	7

## Data Availability

No new data were created or analyzed in this study. Data sharing is not applicable to this article.
